# The E2-Like Conjugation Enzyme Atg3 Promotes Binding of IRG and Gbp Proteins to *Chlamydia*- and *Toxoplasma*-Containing Vacuoles and Host Resistance

**DOI:** 10.1371/journal.pone.0086684

**Published:** 2014-01-17

**Authors:** Arun K. Haldar, Anthony S. Piro, Danielle M. Pilla, Masahiro Yamamoto, Jörn Coers

**Affiliations:** 1 Departments of Molecular Genetics and Microbiology and Immunology, Duke University Medical Center, Durham, NC, United States of America; 2 Department of Microbiology and Immunology, Osaka University, Osaka, Japan; Auburn University, United States of America

## Abstract

Cell-autonomous immunity to the bacterial pathogen *Chlamydia trachomatis* and the protozoan pathogen *Toxoplasma gondii* is controlled by two families of Interferon (IFN)-inducible GTPases: Immunity Related GTPases (IRGs) and Guanylate binding proteins (Gbps). Members of these two GTPase families associate with pathogen-containing vacuoles (PVs) and solicit antimicrobial resistance pathways specifically to the intracellular site of infection. The proper delivery of IRG and Gbp proteins to PVs requires the autophagy factor Atg5. Atg5 is part of a protein complex that facilitates the transfer of the ubiquitin-like protein Atg8 from the E2-like conjugation enzyme Atg3 to the lipid phosphatidylethanolamine. Here, we show that Atg3 expression, similar to Atg5 expression, is required for IRG and Gbp proteins to dock to PVs. We further demonstrate that expression of a dominant-active, GTP-locked IRG protein variant rescues the PV targeting defect of Atg3- and Atg5-deficient cells, suggesting a possible role for Atg proteins in the activation of IRG proteins. Lastly, we show that IFN-induced cell-autonomous resistance to *C. trachomatis* infections in mouse cells depends not only on Atg5 and IRG proteins, as previously demonstrated, but also requires the expression of Atg3 and Gbp proteins. These findings provide a foundation for a better understanding of IRG- and Gbp-dependent cell-autonomous resistance and its regulation by Atg proteins.

## Introduction

Mammalian cells use an expansive network of cell-autonomous defense pathways to combat intracellular pathogens [1]. These defense pathways can be activated by both intrinsic and extrinsic signals. Professional immune cells as well as infected cells produce extrinsic, immune-activating signals in the form of proinflammatory cytokines such as IFNs. Once bound to their cognate receptors, IFNs trigger cell-autonomous immunity through the induction of the “interferome,” a network of more than one thousand IFN-regulated genes [2], [3]. Amongst the most robustly expressed IFN-inducible genes are GTPases [4].

IFN-inducible GTPases can be grouped into four families: Myxovirus-resistance (Mx) proteins, Very Large Inducible GTPases (VLIGs), IRG and Gbp proteins. Whereas the function of VLIG proteins is unknown, Mx, IRG and Gbp proteins have demonstrated roles in host defense [4]. Mx proteins act as antivirals and provide resistance to viruses such as influenza and HIV in humans [5]–[7]. Gbp proteins have also been implicated in controlling intrinsic antiviral immunity; however, they are best characterized for their ability to restrict growth of intracellular bacterial and protozoan pathogens [4]. Similar to Gbp proteins, IRG proteins provide cell-autonomous immunity towards a subset of non-viral pathogens that include the protozoan *Toxoplasma gondii* and the bacterium *Chlamydia trachomatis* [4], [8]–[10]. Both of these pathogens reside within vacuolar compartments known as a parasitophorous or pathogen-containing vacuoles, which we will refer to as PVs. Docking of IRG and Gbp proteins to PVs is essential to contain parasitic growth within IFN-activated cells [9], [11]–[13]. Once recruited to PVs, IFN-inducible GTPases mediate the recruitment of antimicrobial defense modules that include, for example, components of the autophagic machinery [4].

The IRG protein families can be divided into two groups based on the specific P-loop sequence in their nucleotide-binding sites: GKS proteins feature a canonical P-loop sequence (glycine, lysine, serine = GKS) whereas IRGM proteins (also known as GMS proteins) feature a non-canonical P-loop sequence (glycine, methionine, serine = GMS) [14]. In addition to the aforementioned differences in their P-loop sequences and other structural distinctions, GKS and IRGM proteins also differ in their sub-cellular location: whereas IRGM proteins localize to endomembranes and organelles, GKS proteins predominantly reside in the cytosol but translocate to PVs, once a host cell becomes infected with a vacuolar pathogen [15]–[17]. The precise mechanism by which GKS proteins are able to identify PVs as their targets is incompletely understood.

Recently, we were able to demonstrate that GKS proteins identify and target PVs, because PV membranes – in contrast to endomembranes - are devoid of IRGM proteins [18]. IRGM proteins act as guanine dissociation inhibitors (GDIs) for GKS proteins which transition between GDP- and GTP-bound states [19]. GKS proteins in the GTP-bound state form higher order protein oligomers that can bind to PV membranes [18]–[21]. Through transient protein-protein interactions with GDP-bound monomeric GKS proteins, IRGM protein can prevent GKS protein from acquiring GTP, oligomerizing and binding to IRGM-decorated endomembranes [19], [20]. The absence of IRGM proteins from PVs is therefore a prerequisite for GKS activation and membrane binding. However, additional cellular pathways and host factors may influence the efficiency with which GKS proteins target PVs. In support of such a model, the autophagy protein Atg5 was previously identified as a host factor required for the efficient targeting of GKS proteins to PVs [22]–[25].

Eukaryotic cells can modify intracellular membranes by covalently attaching members of the ubiquitin-like protein (Ubl) Atg8 protein family to the lipid phosphatidylethanolamine. Analogous to protein ubiquitination, this reaction is catalyzed by a set of enzymes with E1-, E2- and E3-like activities, termed Atg7, Atg3 and Atg5-Atg12 [26]. In addition to controlling the process of canonical macroautophagy, *Atg* genes have demonstrated roles in regulating additional cellular activities. These activities include, for example, the execution of alternative degradation pathways [27], the initiation of antimicrobial phagocytosis [28] and the inhibition of viral replication complexes [29]. Importantly, execution of these additional pathways frequently depends only on subsets, or *cassettes*, of canonical autophagy proteins [29]. For example, the shrinking of midgut cells during the development of *Drosophila* larvae has been shown to require Atg5 but not the E2-like conjugation enzyme Atg3 [30].

To better understand the role of autophagy-related genes in IFN-driven cell-autonomous immunity, we compared the ability of Atg5- and Atg3-deficient cells to execute IRG-/Gbp-dependent resistance to *T. gondii* and *C. trachomatis* infections. We found that Atg3, similar to Atg5, was required for cell-autonomous resistance and the efficient targeting of both GKS and Gbp proteins to PVs. The requirement for Atg5 and Atg3 in PV targeting could be overcome by expressing a dominant-active, GTP-bound form of the GKS protein Irgb10. These data suggest that Atg3-/Atg5-mediated Ubl lipidation may play a role in promoting GKS protein activation that is independent of the roles of Atg3 and Atg5 in degradative autophagy.

## Materials and Methods

### Host Cell Culture, Bacterial and Protozoan Strains and Infections

MEFs derived from wildtype (WT), *Atg3* ^−/−^, *Atg5* ^−/−^ and *Gbp^chr3^* ^−/−^ mice were previously described [31]–[33]. MEFs and African green monkey kidney Vero cells were cultured in Dulbecco’s modified Eagle’s medium supplemented with 10% heat-inactivated fetal bovine serum (FBS) (Denville and Life Technologies). *C. trachomatis* LGV-L2 were propagated as described [18]. A previously described GFP expression vector was transformed into *C. trachomatis*, as described [34]. GFP-expressing *Toxoplasma gondii* tachyzoites of the type II strain Prugniaud A7 and tachyzoites of the type II ME49 strain were propagated in Vero cells, as described [18] Infections with *C. trachomatis* were performed at a nominal multiplicity of infection of 1–5, as described [18]. For *T. gondii* infections cells were incubated overnight with or without 200 U/ml of IFNγ and asynchronously infected with tachyzoites at a nominal multiplicity of infection of 5–10.

### Immunocytochemistry

Immunocytochemistry was performed essentially as described previously [18]. Cells were washed thrice with PBS, pH 7.4 prior to fixation. Cells were fixed either with methanol or with 3% formaldehyde and 0.025% glutaraldehyde for 20 min at room temperature (RT). In all experiments involving formaldehyde/glutaraldehyde fixation, fixed cells were permeabilized/blocked with 0.05% (v/v) saponin and 2% BSA/PBS (SBP) for 30 min at RT. Then cells were stained with various primary antibodies, followed by Alexa Fluor-conjugated secondary antibodies (Molecular Probes/Invitrogen). Nucleic and bacterial DNA were stained with Hoechst 33258 according to the manufacturer’s protocol. Stained cells were washed with PBS, mounted on microscope slides with FluorSave (Calbiochem) or ProLong Gold (Invitrogen), and allowed to cure overnight. Cells were imaged using either a Zeiss LSM 510 inverted confocal microscope or a Zeiss Axioskop 2 upright epifluorescence microscope. Colocalization of proteins with PVs was quantified in at least 3 independent experiments. In each experiment at least ten randomly selected fields were imaged for each experimental condition and cell type. To determine the frequency with which GKS and Gbp proteins colocalize with PVs, at least one hundred PVs were assessed for each experimental condition and cell type. Differential interference contrast images were used to identify extracellular *T. gondii* tachyzoites. The fraction of Gbp2- or Irgb10-positive vacuoles was determined for each field by dividing the number of Gbp2- or Irgb10-labeled vacuoles by the total number of vacuoles. Colocalization with *C. trachomatis* inclusions was quantified using the identical approach.

### Immunoblotting

Protein samples from whole cell lysates were analyzed by SDS-PAGE and Western blot, as described previously [18] Blots were probed with primary antibodies specific for: Atg3 (Abcam), Atg5 (Novus Biologicals), p62/SQSTM1 (MBL international), LC3 (MBL international), Gbp2, β-actin (Sigma). Binding of secondary HRP-labeled goat-anti-rabbit or goat-anti-mouse antibodies (Thermo Scientific) was analyzed using SuperSignal^(R)^ West Pico or West Femto Chemiluminescent Substrate (Thermo Scientific).

### Antibodies

The primary antibodies used included anti-Gbp2 at 1∶1000 [18], anti-Irga6 rabbit polyclonal antibody 765B0 [35] at 1∶50000; anti-Irgb10 rabbit polyclonal antiserum [9] at 1∶1000; anti-Irgb6 rabbit polyclonal antisera [35] at 1∶1000; FITC-labeled mouse monoclonal anti-*C. trachomatis* MOMP [9] at 1∶200; rabbit anti-IncG [36] at 1∶50; anti-p62/SQSTM1 rabbit polyclonal antibody (MBL International) at 1∶500; anti-LC3 rabbit polyclonal antibody (MBL International) at 1∶1000; anti-Atg3 rabbit monoclonal antibody (Abcam) at 1∶10000; anti-Atg5 rabbit polyclonal antibody (Novus Biologicals) at 1∶500; and anti-*T. gondii* rabbit polyclonal antibody (Biogenex) at 1∶500.

### Cell Transfection and Transduction

An Irgb10-GFP expression construct and the Irgb10^S82N^ and Irgb10^K81A^ mutants have been previously described [18]. MEFs were transfected using Attractene (Qiagen) following the manufacturers’ instructions.

### Quantitative PCR

Total nucleic acid was prepared from trypsinized cell pellets using the QIAamp DNA Mini Kit from Qiagen (Valencia, CA USA). Samples were then subjected to singleplex qPCR on an ABI 7000 Sequence Detection System to assess the amount of 16s *Chlamydia* and GAPDH host DNA in the sample. *Chlamydia* 16s DNA was detected through use of the following primer sequences, essentially as described [37]: 16sforward primer 5′-GGA GGC TGC AGT CGA GAA TCT-3′, reverse primer 5′-TTA CAA CCC TAG AGC CTT CAT CAC A-3′, and dual-labeled probe 5′-[6-FAM]-TCG TCA GAC TTC CGT CCA TTG CGA-[TAMRA]-3′. Mouse GAPDH DNA was detected using the Rodent GAPDH Control Reagent Kit from Applied Biosystems (Foster City, CA, USA). Standard curves were generated in parallel from known amounts of *C. trachomatis* and murine DNA, and these curves were used to calculate the amount (pg) of *Chlamydia* DNA per unit mass (µg) of mouse DNA in the samples.

### Statistical Analysis

Results are represented as means ± SD. The unpaired two-tailed Student’s t test was used to determine the statistical significance of the experimental data where p≤0.05 was considered significant.

## Results

### Atg3 and Atg5 Promote the Delivery of IFN-inducible GTPases to *T. gondii* PVs and *C. trachomatis* Inclusions

**Figure 1:**
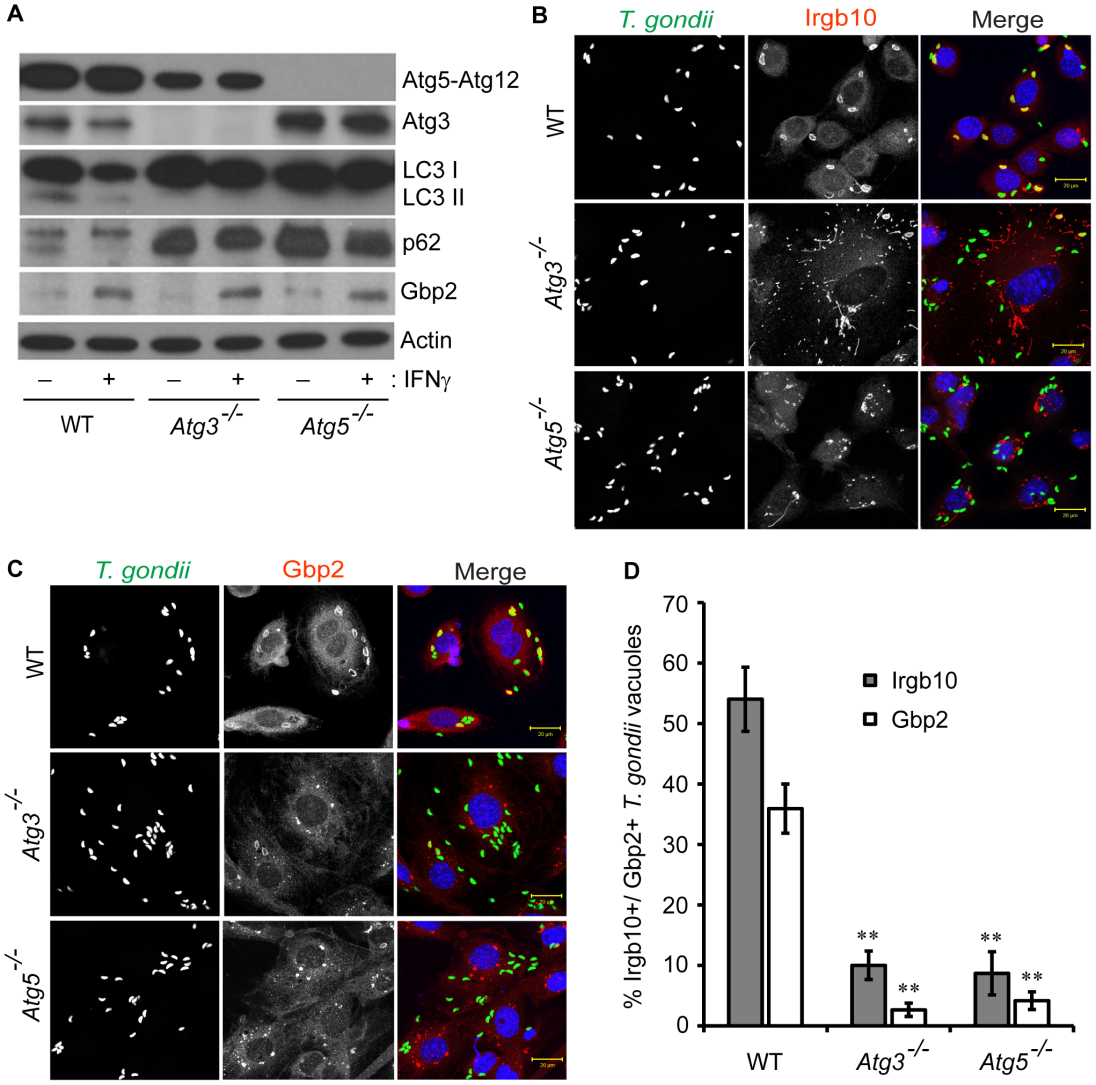
Atg3 and Atg5 promote the delivery of IFN-inducible GTPases to *T. gondii* PVs. (**A**) Wildtype (WT), *Atg3^−/−^* and *Atg5^−/−^* MEFs were treated overnight with 200 U/ml of IFNγ or were left untreated. Protein extracts were analyzed by Western blotting using antibodies reactive to Atg3, Atg5, p62, LC3, Gbp2 and actin. (**B–D**) WT, *Atg3^−/−^* and *Atg5^−/−^* MEFs were treated overnight with 200 U/ml of IFNγ prior to infections. Localization of endogenous Irgb10 (**B and D**) and Gbp2 (**C and D**) to *T. gondii* PVs was monitored at 0.5 hpi. Data are representative of three independent experiments (**, p<0.005 relative to wildtype). Error bars represent standard deviations. Representative confocal images of *T. gondii*-infected MEFs are shown in B and C.

Targeting of GKS proteins to *T. gondii* PVs requires the expression of the autophagy protein Atg5 [24], [25]. Autophagy is controlled by two Ubl conjugation systems: the first system conjugates the Ubl Atg12 to Atg5 and the second system conjugates the Ubl Atg8 (i.e. LC3 and its paralogs in mammalian cells) to lipids [26]. Only the Atg8 but not the Atg12 conjugation system requires the E2-like conjugating enzyme Atg3 and accordingly Atg5-Atg12 conjugates are still formed in *Atg3* ^−/−^ cells (Figure 1A). As expected, we observed that autophagy-deficient *Atg3* ^−/−^ cells and *Atg5* ^−/−^ cells did not generate lipidated LC3 (LC3-II) and instead accumulated p62 protein, a known substrate of the autophagic degradation pathway (Figure 1A).

To determine whether the formation of Atg5-Atg12 conjugates was sufficient to direct GKS proteins to *T. gondii* PVs, we monitored the localization of the GKS protein Irgb10 in *Atg3* ^−/−^ MEFs. We found that colocalization of Irgb10 with *T. gondii* PVs was diminished in *Atg3* ^−/−^ cells relative to wildtype cells (Figure 1B). Colocalization of the Gbp protein Gbp2 with *T. gondii* PVs was also reduced in *Atg3* ^−/−^ cells (Figure 1C). Overall the colocalization of Irgb10 and Gbp2 with *T. gondii* was reduced to similar levels in *Atg3* ^−/−^ and *Atg5* ^−/−^ MEFs (Figure 1D), suggesting that both of the two Ubl conjugation systems controlling autophagy are critical for the delivery and/or attachment of IFN-inducible GTPases to *T. gondii* PVs.

**Figure 2:**
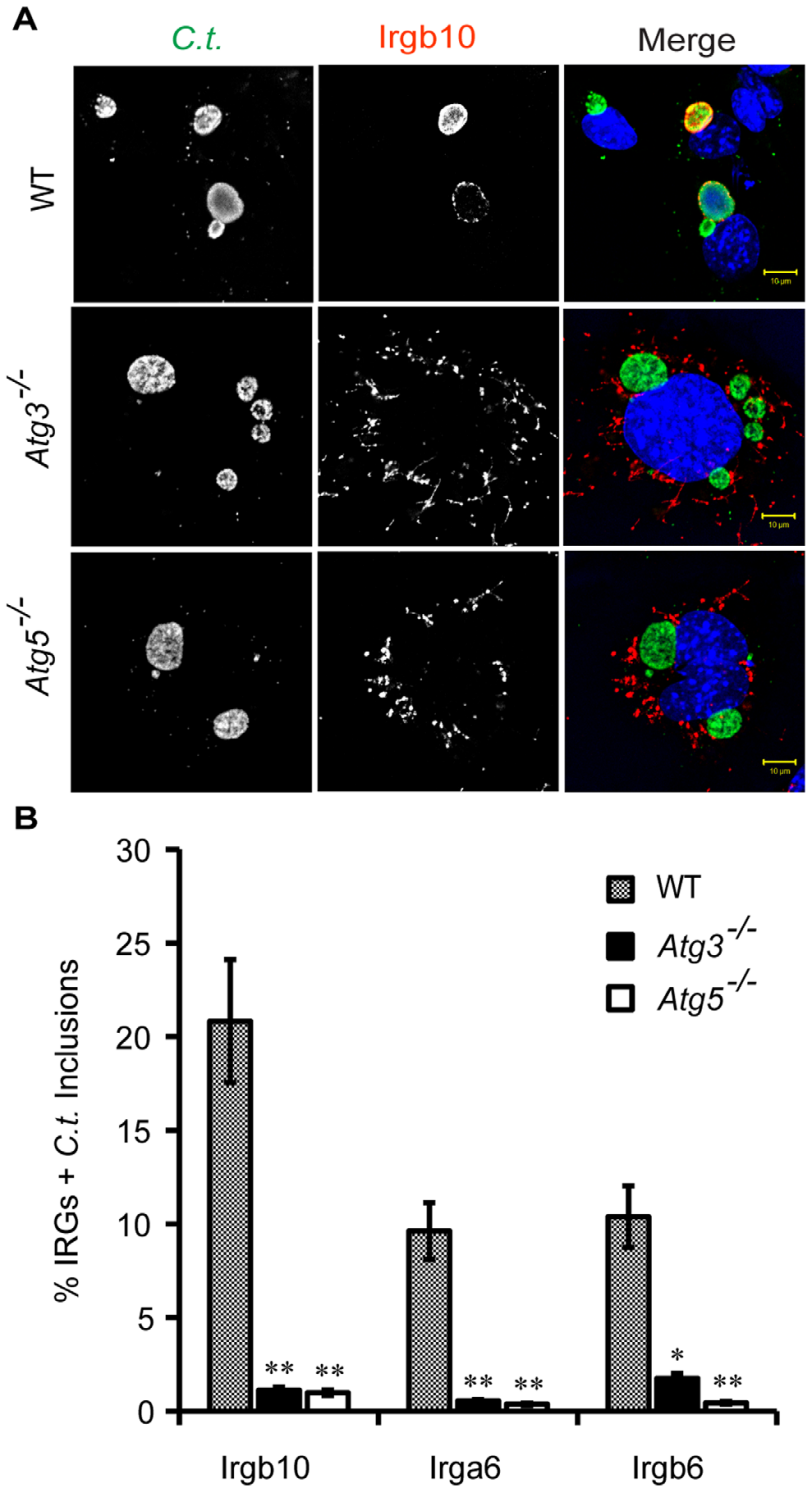
Atg3 and Atg5 promote the delivery of GKS proteins to *C. trachomatis* inclusions. (**A and B**) WT, *Atg3^−/−^* and *Atg5^−/−^* MEFs were infected with *C. trachomatis* and treated with 200 U/ml of IFNγ at 3 hpi. Cells were fixed at 20 hpi and stained with anti-*C. trachomatis* MOMP, anti-Irgb10, anti-Irga6, anti-Irgb6 and Hoechst. Representative staining with anti-Irgb10 is shown. (**B**) Colocalization of Irgb10, Irga6 and Irgb6 with inclusions in WT, *Atg3^−/−^* & *Atg5^−/−^* MEFs was quantified as described in Materials and Methods. The data are representative of three independent experiments. Error bars represent standard deviations. Statistical significance of group values relative to wildtype is shown (*, p<0.05, **, p<0.005).

**Figure 3:**
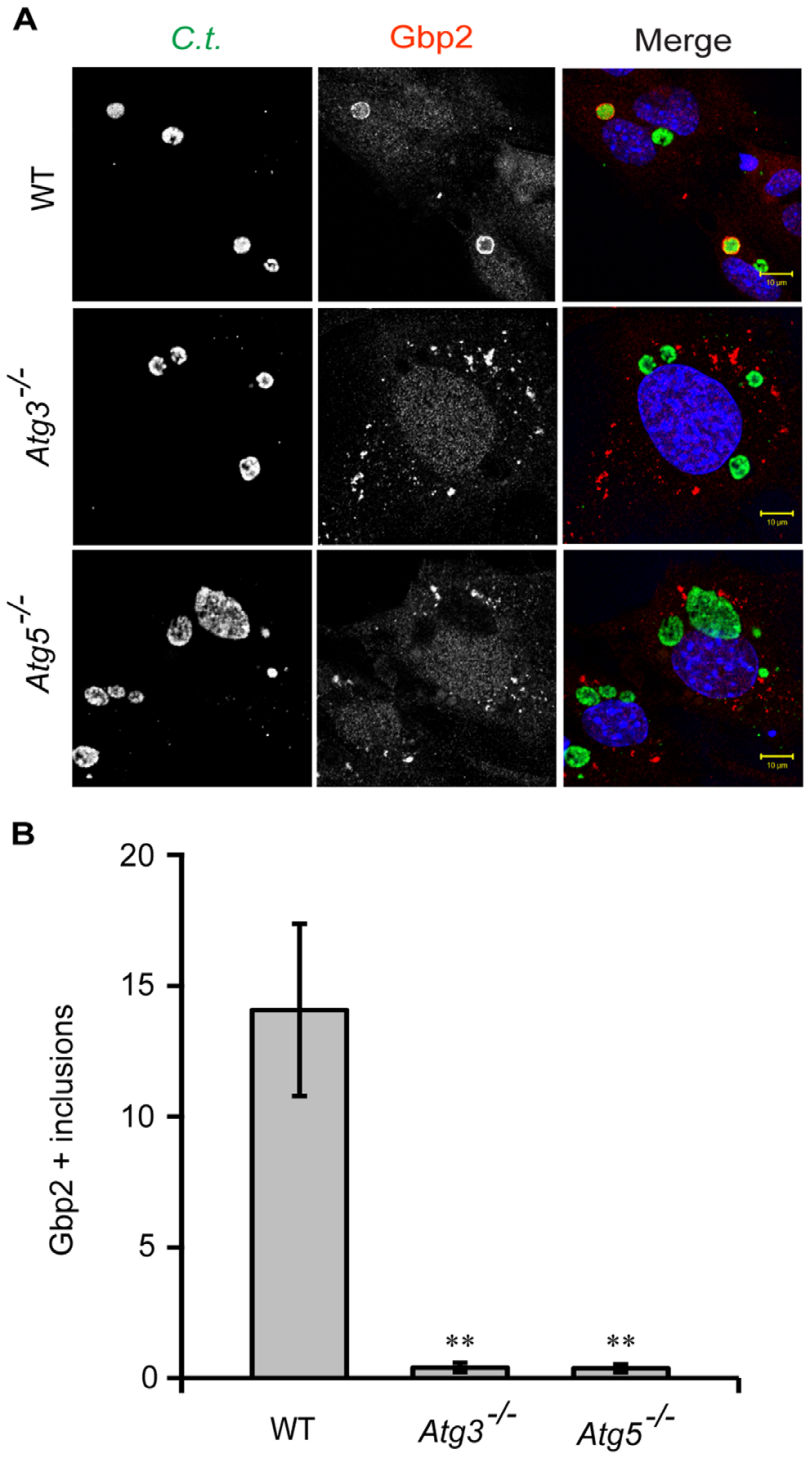
Atg3 and Atg5 promote the efficient delivery of Gbp2 to *C. trachomatis* inclusions. (**A**) WT, *Atg3^−/−^* & *Atg5^−/−^* MEFs were infected with *C. trachomatis* and treated with IFNγ at 3 hpi. Cells were fixed at 20 hpi and stained with anti-*C. trachomatis* MOMP, anti-Gbp2 and Hoechst. Representative images are shown. (**B**) Colocalization of Gbp2 with inclusions in WT, *Atg3^−/−^* and *Atg5^−/−^* MEFs was quantified as described in Materials and Methods. Error bars represent standard deviations of three independent experiments. Statistical significance of group values relative to wildtype is shown (**, p<0.005).

As previously reported, targeting of GKS proteins to *C. trachomatis* PVs, commonly referred to as *inclusions*, also requires Atg5 expression [22]. Here, we observed that the colocalization of the GKS proteins Irgb10, Irga6 and Irgb6 with inclusions was not only dependent on Atg5 but also on Atg3 expression (Figure 2 and Figure S1). Additionally, we found that endogenous Gbp2 was largely absent from *C. trachomatis* inclusions in both *Atg5* ^−/−^ and *Atg3* ^−/−^ cells (Figure 3). Collectively, these data show that Atg3 and Atg5 are both required for the efficient delivery of GKS and Gbp proteins to PVs formed by two distinct pathogens.

### The GTP-locked Irgb10-K81 Mutant Efficiently Targets *C. trachomatis* Inclusions and *T. gondii PVs* in Atg3- and Atg5-deficient Cells

**Figure 4:**
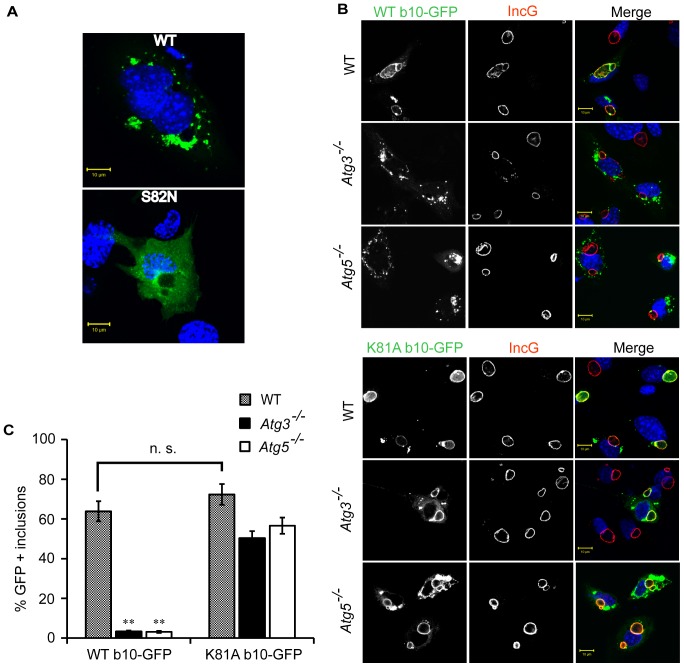
GTP-locked Irgb10^K81A^ mutant but not wildtype Irgb10 targets *C. trachomatis* inclusions in *Atg3*- and *Atg5*-deficient cells with high efficiency. (**A**) *Atg5^−/−^* MEFs were transfected with GFP-fusion constructs expressing either wildtype Irgb10 (WT) or the Irgb10^S82N^ mutant that is deficient of GTP binding. Cells were subsequently infected with *C. trachomatis* and treated with 200 U/ml of IFNγ at 3 hpi. Fixed cells were stained with Hoechst. Representative images are shown. (**B**) WT, *Atg3^−/−^* & *Atg5^−/−^* MEFs were transfected with the indicated constructs. Cells were infected with *C. trachomatis* and treated with IFNγ at 3 hpi. Cells were fixed at 20 hpi and stained for the *C. trachomatis* inclusion membrane marker IncG as well as DNA (Hoechst). Representative images are shown. (**C**) Graphical representation of the frequency at which WT Irgb10 and the Irgb10^K81A^ mutant colocalize with inclusions. Average values ± SD of three independent experiments are shown. Differences in the targeting frequency for WT Irgb10 and Irgb10^K81A^ to inclusions were evaluated for statistical significance (**, p<0.005).

The mechanism by which Atg5 controls the targeting of GKS proteins to PVs is unknown. One previously proposed model predicts that Atg5-dependent autophagy is required to maintain a pool of soluble, cytosolic GKS proteins available for PV targeting. This model is based on the observation that Atg5-deficient cells accumulate GTP-bound GKS protein aggregates in the cytosol [25] and the prediction that these protein aggregates would sequester GKS proteins away from PV targeting. To directly test whether GTP binding was necessary for the formation of GKS protein aggregates in *Atg5* ^−/−^ cells, we took advantage of the previously described Irgb10^S82N^ mutant that is deficient for GTP binding [18]. We found that wildtype Irgb10 GFP-fusion proteins formed aggregates in *Atg5* ^−/−^ cells but Irgb10^S82N^ GFP-fusion proteins did not (Figure 4A). These data indicate that GTP acquisition is required for the formation of Irgb10 protein aggregates in autophagy-deficient cells, as previously suggested [25]. We also observed the formation of Irgb10 protein aggregates in *Atg3* ^−/−^ cells (Figures 1 and 2), further supporting a possible model in which a defect in autophagic clearance of cytosolic GTP-bound GKS protein oligomers results in the formation of GKS protein aggregates. However, these results failed to determine whether or not aggregate formation causes the prevalent PV targeting defect observed in *Atg5* ^−/−^ and *Atg3* ^−/−^ cells.

Irgb10 protein aggregates form not only in *Atg5* ^−/−^ and *Atg3* ^−/−^ but also in *Irgm1/m3* cells. However, in *Irgm1/m3* cells Irgb10 targets *C. trachomatis* inclusions with high efficiency [18]. Therefore, Irgb10 aggregate formation seemed unlikely to be the cause for the Irgb10 targeting defect observed in *Atg5* ^−/−^ and *Atg3* ^−/−^ cells. Hence, we considered an alternative model in which Atg3 and Atg5 would play a more direct role in facilitating Irgb10 binding to PV membranes. Because the transition of Irgb10 into the GTP-bound, active state is a prerequisite for Irgb10 PV membrane binding [18], we considered that Atg3 and Atg5 could be involved in promoting Irgb10 activation. Therefore, we hypothesized that a GTP-locked, constitutively active Irgb10 mutant should target *C. trachomatis* independently of Atg3 and Atg5. To test this hypothesis, we ectopically expressed GFP-fusions of wildtype Irgb10 and the previously described GTP-locked mutant Irgb10^K81A^ [18] and monitored their subcellular localization. We found that both Irgb10 variants localized to inclusions with comparable frequency in wildtype cells (Figures 4B and 4C). In *Atg3*- and *Atg5*-deficent cells, however, ectopically expressed Irgb10, similar to endogenous Irgb10, failed to localize to inclusions (Figures 4B and 4C). In contrast to wildtype Irgb10, the GTP-locked Irgb10^K81A^ mutant localized to inclusions formed in *Atg5* ^−/−^ and *Atg3* ^−/−^ cells at high frequency (Figure 4B and 4C). Irgb10^K81A^ but not wildtype Irgb10 similarly localized to *T. gondii* PVs in *Atg5* ^−/−^ and *Atg3* ^−/−^ cells (Figure S2), demonstrating that constitutive-active Irgb10 can target PVs formed by different pathogens independently of Atg5 and Atg3. Colocalization of Irgb10^K81A^ with PVs occurred in spite of Irgb10 protein aggregate formation (Figure 4B and Figure S2). Therefore, the formation of GTP-bound Irgb10 aggregates is not sufficient to explain the failure of endogenous Irgb10 to target inclusions in *Atg5* ^−/−^ and *Atg3* ^−/−^ cells. Instead, our data suggest a possible involvement for Atg3 and Atg5 in promoting the transition of Irgb10 into the GTP-bound active state.

### Atg3 and Atg5 are Required for IFNγ-induced Cell-autonomous Resistance to *C. trachomatis* Infections

**Figure 5:**
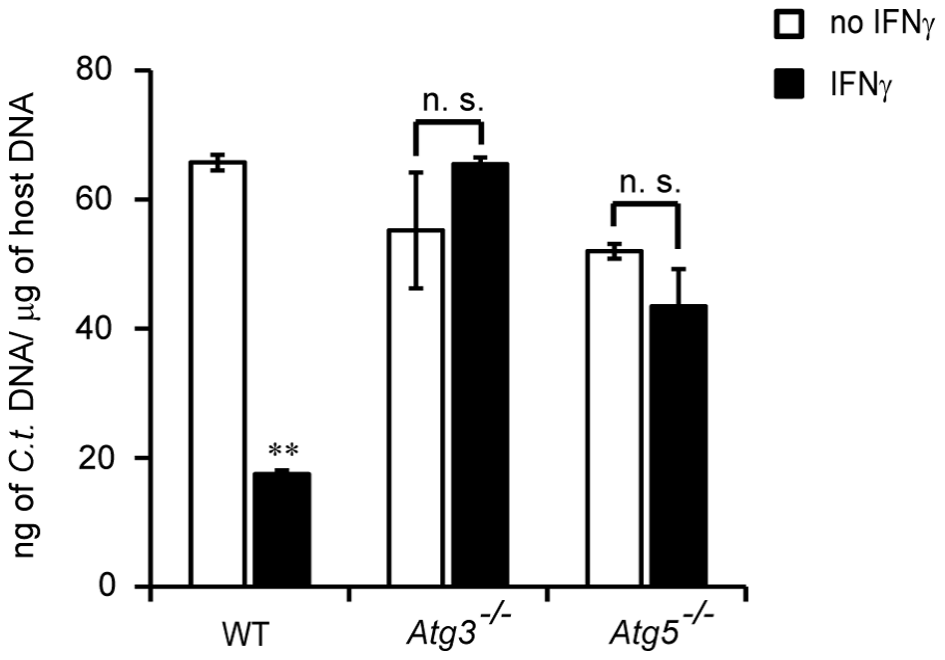
*Atg3*- and *Atg5*-deficient cells exert minimal IFNγ-induced cell-autonomous resistance to *C. trachomatis*. WT, *Atg3^−/−^* and *Atg5^−/−^* MEFs were either treated overnight with 200 U/ml of IFNγ or remained untreated. Cells were then infected with *C. trachomatis* for 24 h and total DNA was harvested for each biological sample. Chlamydial DNA was quantified by qPCR as described in Methods and Materials. Data are representative of three independent experiments. Statistical significance of group values between untreated and IFNγ-treated cells is shown (**, p<0.005; n. s. – not significant).

Because Atg3- and Atg5-deficient cells failed to efficiently deliver GKS proteins like Irgb10 to inclusions, we monitored the ability of these cells to restrict intracellular chlamydial growth following IFNγ activation. As our wildtype control we used *Atg3* ^+/+^ MEFs derived from *Atg3* ^−/−^embryo littermates. Whereas IFNγ-activated wildtype cells significantly reduced bacterial burden relative to untreated controls, *Atg3* ^−/−^ cells had lost their ability to restrict chlamydial growth (Figure 5). As shown previously [22], we also observed a defect in *Atg5* ^−/−^ cells to contain bacterial burden upon IFNγ activation (Figure 5), demonstrating the importance for both Atg3 and Atg5 in cell-autonomous immunity to *C. trachomatis* infections.

### A Cluster of Gbp Proteins on Mouse Chromosome 3 Provides Cell-autonomous Resistance to *C. trachomatis* Infections

**Figure 6:**
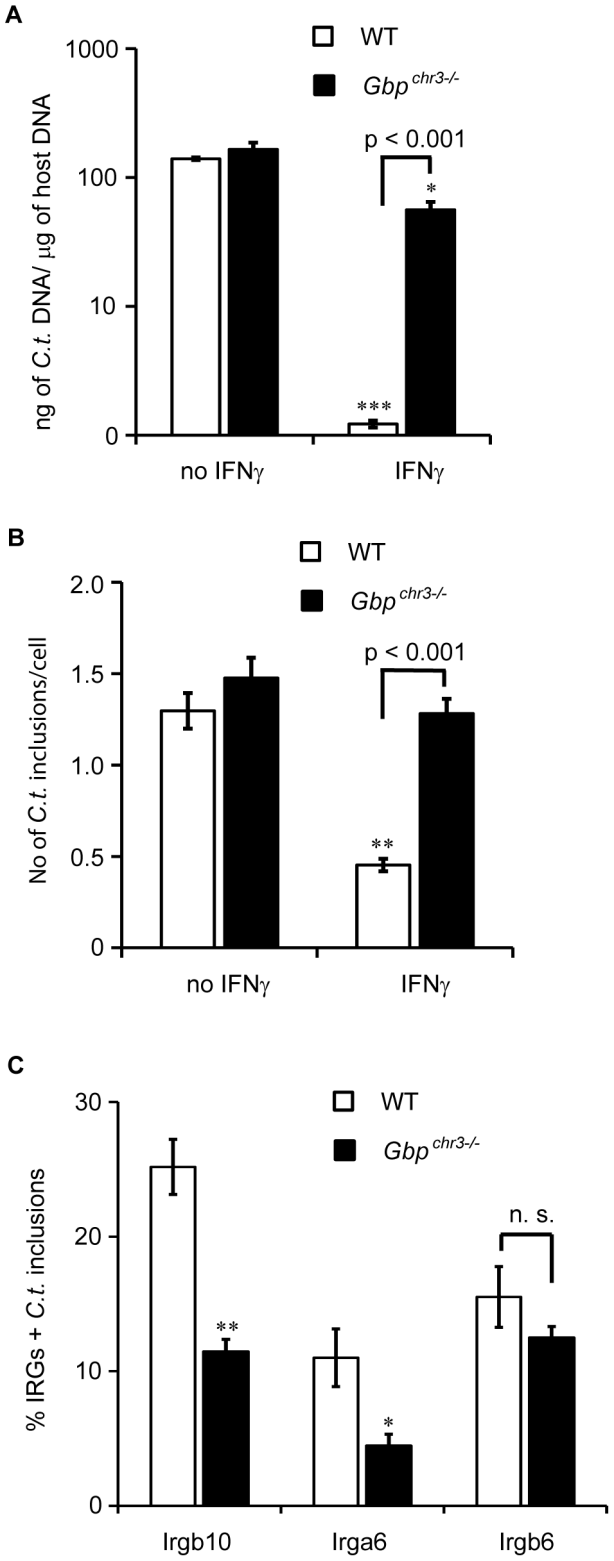
A cluster of Gbp proteins on mouse chromosome 3 provides cell-autonomous resistance to *C. trachomatis* infections. (**A**) WT and *Gbp^chr3−/−^* MEFs were either treated overnight with 200 U/ml of IFNγ or left untreated and subsequently infected with *C. trachomatis* for 24 h. Bacterial burden was assessed by qPCR as described in Methods and Materials. Data are representative of three independent experiments. Statistical significance of group values between untreated and IFNγ-treated cells is shown (*, p<0.05; ***, p<0.005). (**B**) WT and *Gbp^chr3−/−^* MEFs were infected with GFP-expressing *C. trachomatis*, left untreated or treated with IFNγ at 3 hpi. Cells were fixed at 20 hpi and stained with Hoechst. The number of *C. trachomatis* inclusions per cell was measured. Data are representative of three independent experiments. Graph represents average values ± SD. Statistical significance of group values between untreated and IFNγ-treated cells is shown (**, p<0.005). (**C**) WT and *Gbp^chr3−/−^* MEFs were infected with GFP-expressing *C. trachomatis* and treated with IFNγ at 3 hpi. Cells were fixed at 20 hpi and stained with anti-Irgb10, anti-Irga6, anti-Irgb6 and Hoechst. Colocalization of Irgb10, Irga6 and Irgb6 with inclusions in WT, *Atg3^−/−^* & *Atg5^−/−^* MEFs was quantified as described in Materials and Methods. Error bars represent standard deviations of three independent experiments. Statistical significance of group values relative to wildtype is shown (*, p<0.05, **, p<0.005).

We next asked whether the failure of *Atg5* ^−/−^ and *Atg3* ^−/−^ cells to restrict chlamydial growth upon IFNγ activation could in part be caused by the inability of these cells to target Gbp proteins to inclusions and thus to execute Gbp-mediated cell-autonomous immunity. To test this hypothesis, we obtained *Gbp^chr3^* ^−/−^ MEFs that are deficient for a cluster of mouse *Gbp* genes encoded on mouse chromosome 3 [33]. This cluster encompasses the genes *Gbp1*, *Gbp2*, *Gbp3*, *Gbp5* and *Gbp7*. We stimulated *Gbp^chr3^* ^−/−^ and littermate-derived control MEFs with IFNγ over night and subsequently infected these cells with *C. trachomatis* for 24 hours. While IFNγ activation lowered bacterial burden in control MEFs by approximately 2 logs, IFNγ-activated *Gbp^chr3^* ^−/−^ MEFs reduced bacterial burden minimally compared to wildtype cells (Figure 6A). To gain a better understanding of the kinetics of Gbp-mediated cell-autonomous immunity towards *C. trachomatis*, we activated MEFs with IFNγ at 3 hours post-infection (hpi). We found that immune activation at 3 hpi resulted in a significant decrease in the number of inclusions in wildtype MEFs but failed to reduce the number of inclusions in *Gbp^chr3^* ^−/−^ MEFs (Figure 6B). These data suggest that Gbp proteins help execute resistance pathways that can target established *C. trachomatis* inclusions.

Because it was previously reported that Gbp proteins augment the colocalization of GKS proteins with *T. gondii* PVs [33], we explored whether Gbp proteins also promote GKS protein association with *C. trachomatis* inclusions. We found that the frequency of Irgb10 and Irga6 but not Irgb6 colocalization with inclusions was moderately reduced in *Gbp^chr3^* ^−/−^ cells (Figure 6C). Therefore, failure to retain GKS proteins at the inclusion may in part explain the loss of cell-autonomous immunity in *Gbp^chr3^* ^−/−^ cells.

## Discussion

Amongst the most abundantly expressed IFN-inducible proteins are GTPases of the IRG and Gbp families. The robust expression of these GTPases in immune-activated host cells immediately suggested a potential role for these proteins in providing resistance to infections. This obvious assumption was convincingly confirmed with the engineering and initial characterization of mice deficient for the IRG genes *Irgm1* and *Irgm3* [10], [38]. Mice lacking either *Irgm1* or *Irgm3* individually or both simultaneously were found to be more susceptible to infections with the protozoan pathogen *T. gondii* as well as the bacterial pathogen *C. trachomatis* [9], [10], [38]–[40]. More recently, genetic deletions of individual *Gbp* genes as well as the deletion of the *Gbp* gene cluster on chromosome 3 demonstrated the importance of this second GTPase family in resistance to *T. gondii* infections [25], [33], [41], [42]. Here, we demonstrate that Gbp proteins also provide resistance to *C. trachomatis* infections in mouse cells. Because human Gbp proteins were previously shown to restrict intracellular chlamydial replication [43], [44], our data suggest that Gbp-mediated immunity directed against *C. trachomatis* may be conserved between mice and humans.

Both in human and mouse cells Gbp proteins associate with *C. trachomatis* inclusion membranes [18], [43], [44]. Similar to the behavior of Gbp proteins, the GKS group of IRG proteins binds to inclusion membranes as well as PV membranes surrounding *T. gondii* [9], [22], [45], [46]. Gbp and GKS proteins colocalize at PVs [33], [47] and several lines of evidence indicate that Gbp and GKS proteins promote each other’s association with PVs [18], [33]. In further support of these previous results, we show here that a subset of GKS proteins target inclusions with diminished efficiency in *Gbp^chr3^* ^−/−^ cells. Although the mechanism by which these two protein families influence one another’s subcellular localization is currently unknown, these observations may suggest the existence of one or more PV targeting pathways that are shared between GKS and Gbp proteins.

The concept that Gbp and GKS proteins may be recruited to PVs by overlapping or identical cellular pathways is further supported by previous reports demonstrating that GKS and Gbp proteins both require Atg5 expression in order to efficiently associate with PVs [22]–[25]. While the importance for Atg5 in directing Gbp and GKS proteins to PVs is now well established, the mechanism by which Atg5 promotes the association of IFN-inducible GTPases with intracellular pathogens has remained largely unexplored. Here, we demonstrate that Gbp and GKS translocation to PVs also requires Atg3, the E2-like conjugation enzyme essential for the lipidation of Atg8 proteins. These data therefore indicate that Atg8-lipidation is essential to target members of both families of IFN-inducible GTPases to PVs.

The mammalian Atg8 protein family consists of seven homologs that can be grouped into three subfamilies: LC3, GABARAP and GATE-16. Similar to LC3, GABARAP and GATE-16 exist as both non-lipidated and lipidated forms, of which the latter ones associate with autophagosomes [48]. Once covalently linked to lipids, different Atg8 homologs appear to fulfill partly distinct, non-redundant functions in cargo recognition, autophagosome biogenesis and autophagosome maturation [48]. In addition to their roles in the execution of autophagy, Atg8 proteins have also been implicated in non-autophagic functions, which include intra-Golgi transport and unconventional secretion of proinflammatory cytokines [49]–[51]. At least some of these “alternative,” non-autophagic functions are also dependent on the Atg8 lipidation machinery [49]. Therefore, unsuspected phenotypes observed in *Atg5* ^−/−^ and *Atg3* ^−/−^ cells should be interpreted with caution, as they may not always be the result of a defective autophagic process. Indeed, as previously noted, the failure of *Atg5* ^−/−^ cells to deliver the GKS protein to PVs is not satisfactory explained with a defect in autophagy [24].

IFN activation of autophagy-deficient cells results in the formation of cytosolic GKS protein aggregates [24]. These aggregates appear to be composed of GTP-bound proteins, as detected by the use of a conformation-specific anti-Irga6 antibody [25]. In agreement with these previous observations, we show that GTP acquisition is necessary for aggregate formation in *Atg5* ^−/−^ cells. Similar to *Atg5* ^−/−^ cells, IRGM-deficient cells also accumulate aggregate-like GKS punctae that are composed of GTP-bound proteins [18], [19], [35]. However, critically distinct from the cytosolic GKS aggregates formed in *Atg5* ^−/−^ cells [25], GKS punctae formed in IRGM-deficient cells are membrane bound [18]. These observations imply distinct functions for Atg5 and IRGM proteins in regulating GKS activities. Strong experimental evidence indicates that membrane-bound IRGM proteins act as GDIs for GKS proteins and thereby block GKS binding to IRGM-decorated membranes [18], [19]. In the absence of IRGM proteins, GKS proteins can bind to these IRGM-stripped membranes [18]. In *Atg5* ^−/−^ cells on the other hand, GKS aggregates form in the cytosol [25], suggesting that the presence of Atg5 favors the activation of GKS proteins at membranes. Atg5 may do this in two ways: 1) by clearing GTP-bound GKS proteins from the cytosol and 2) by retaining GKS proteins at Atg8-decorated membranes and promoting GKS protein activation at these target membranes. In this study we provide data in support of a possible role for Atg5 and Atg3 in GKS protein activation. We show that the constitutive active, GTP-locked mutant form of Irgb10, Irgb10^K81A^, colocalizes with *C. trachomatis* inclusions and *T. gondii* PVs in *Atg5* ^−/−^ and *Atg3* ^−/−^ cells. The substantial rescue of PV targeting by Irgb10^K81A^ is not merely the result of protein overexpression, since overexpression of wildtype Irgb10 fails to target PVs in the absence of Atg3 and Atg5. Because Irgb10^K81A^ is no longer strictly dependent on Atg3 and Atg5 as cofactors for PV targeting, we propose a possible role for Atg3 and Atg5 in GKS protein activation. Although Atg3 and Atg5 may mediate such an activation step directly, it appears more likely that lipidated Atg8 proteins are required for tethering GKS proteins to PV membranes resulting in GKS activation at the target membrane. Future studies will address whether one or more specific Atg8 proteins are required for GKS targeting to PVs.

## Supporting Information

Figure S1 **Atg3 and Atg5 promote the delivery of GKS proteins Irga6 and Irgb6 to** ***C. trachomatis*** **inclusions.** WT, *Atg3^−/−^* and *Atg5^−/−^* MEFs were infected with *C. trachomatis* and treated with 200 U/ml of IFNγ at 3 hpi. Cells were fixed at 20 hpi and stained with Hoechst, anti-*C. trachomatis* MOMP, and anti-Irga6 or anti-Irgb6, respectively. Confocal immunofluorescence images are shown.
(TIF)

Figure S2 **GTP-locked Irgb10^K81A^ mutant but not wildtype Irgb10 targets** ***T. gondii*** **PVs efficiently in** ***Atg3*** **- and** ***Atg5*** **-deficient cells.** **(A)** WT, *Atg3^−/−^* & *Atg5^−/−^* MEFs were transfected with the indicated constructs and treated with 200 U/ml of IFNγ overnight. Cells were infected with the *T. gondii* type II strain ME49 for 3 hours and stained with a polyclonal anti-*T. gondii* antibody as well as Hoechst. Representative images are shown. **(B)** Graphical representation of the frequency at which WT Irgb10 and the Irgb10^K81A^ mutant colocalize with *T. gondii* PVs. Average values ± SD of three independent experiments are shown. Differences in the targeting frequency for WT Irgb10 and Irgb10^K81A^ to inclusions were evaluated for statistical significance (*, p<0.05).
(TIF)
