# Supplementary figures and images for: The E2-Like Conjugation Enzyme Atg3 Promotes Binding of IRG and Gbp Proteins to Chlamydia- and Toxoplasma-Containing Vacuoles and Host Resistance
Source: PLoS One. 2014 Jan 17;9(1):e86684. doi: 10.1371/journal.pone.0086684 (PMC3895038; doi:10.1371/journal.pone.0086684)

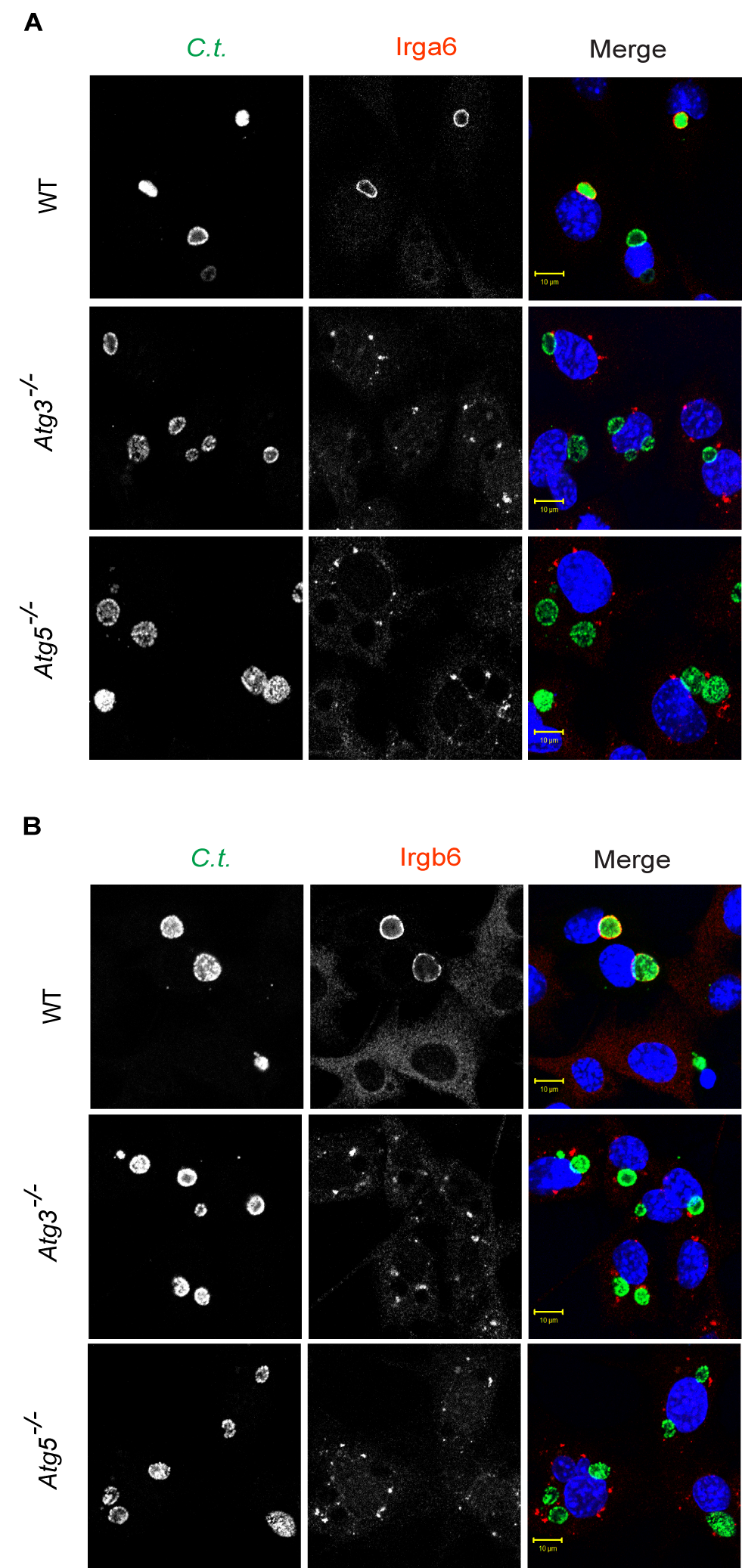

Supplement: Figure S1 — Atg3 and Atg5 promote the delivery of GKS proteins Irga6 and Irgb6 to C. trachomatis inclusions. WT, Atg3−/− and Atg5−/− MEFs were infected with C. trachomatis and treated with 200 U/ml of IFNγ at 3 hpi. Cells were fixed at 20 hpi and stained with Hoechst, anti-C. trachomatis MOMP, and anti-Irga6 or anti-Irgb6, respectively. Confocal immunofluorescence images are shown. (TIF) [file pone.0086684.s001.tif]

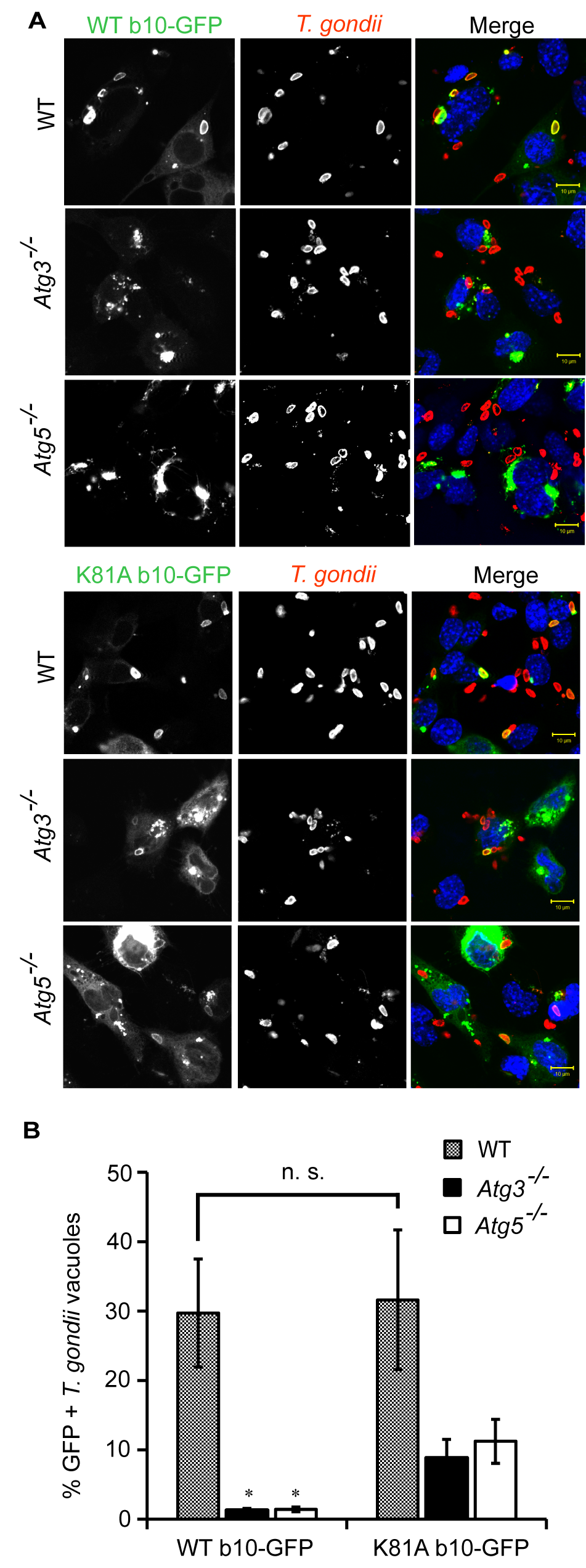

Supplement: Figure S2 — GTP-locked Irgb10K81A mutant but not wildtype Irgb10 targets T. gondii PVs efficiently in Atg3 - and Atg5 -deficient cells. (A) WT, Atg3−/− & Atg5−/− MEFs were transfected with the indicated constructs and treated with 200 U/ml of IFNγ overnight. Cells were infected with the T. gondii type II strain ME49 for 3 hours and stained with a polyclonal anti-T. gondii antibody as well as Hoechst. Representative images are shown. (B) Graphical representation of the frequency at which WT Irgb10 and the Irgb10K81A mutant colocalize with T. gondii PVs. Average values ± SD of three independent experiments are shown. Differences in the targeting frequency for WT Irgb10 and Irgb10K81A to inclusions were evaluated for statistical significance (*, p<0.05). (TIF) [file pone.0086684.s002.tif]
